# A Random Forest-Based Accuracy Prediction Model for Augmented Biofeedback in a Precision Shooting Training System

**DOI:** 10.3390/s20164512

**Published:** 2020-08-12

**Authors:** Junqi Guo, Lan Yang, Anton Umek, Rongfang Bie, Sašo Tomažič, Anton Kos

**Affiliations:** 1 School of Artificial Intelligence, Beijing Normal University, Beijing 100875, China; guojunqi@bnu.edu.cn (J.G.); yanglan@mail.bnu.edu.cn (L.Y.); 2 Engineering Research Center of Intelligent Technology and Educational Application, Ministry of Education, Beijing 100875, China; 3 Academy of Broadcasting Planning, National Radio and Television Administration(NRTA), Beijing 100866, China; 4 Faculty of Electrical Engineering, University of Ljubljana, Tržaška Cesta 25, 1000 Ljubljana, Slovenia; anton.umek@fe.uni-lj.si (A.U.); saso.tomazic@fe.uni-lj.si (S.T.); anton.kos@fe.uni-lj.si (A.K.)

**Keywords:** precision shooting, accuracy prediction model, augmented feedback, random forest

## Abstract

In the military, police, security companies, and shooting sports, precision shooting training is of the outmost importance. In order to achieve high shooting accuracy, a lot of training is needed. As a result, trainees use a large number of cartridges and a considerable amount of time of professional trainers, which can cost a lot. Our motivation is to reduce costs and shorten training time by introducing an augmented biofeedback system based on machine learning techniques. We are designing a system that can detect and provide feedback on three types of errors that regularly occur during a precision shooting practice: excessive hand movement error, aiming error and triggering error. The system is designed to provide concurrent feedback on the hand movement error and terminal feedback on the other two errors. Machine learning techniques are used innovatively to identify hand movement errors; the other two errors are identified by the threshold approach. To correct the excessive hand movement error, a precision shot accuracy prediction model based on Random Forest has proven to be the most suitable. The experimental results show that: (1) the proposed Random Forest (RF) model achieves the prediction accuracy of 91.27%, higher than any of the other reference models, and (2) hand movement is strongly related to the accuracy of precision shooting. Appropriate use of the proposed augmented biofeedback system will result in a lower number of rounds used and shorten the precision shooting training process.

## 1. Introduction

Accuracy is the most important parameter in precision shooting. To achieve the desired level of shooting accuracy, trainees have to practice a lot, which takes a lot of rounds (cartridges) and time. Advances in science and technology today provide the means to improve and develop training techniques. In recent years, many sensors such as accelerometers [1,2], gyroscopes [3,4], and pressure sensors [5,6] have been widely used in various sports activities. For example, ultrasound sensors, which represent a reasonable compromise between cost and accuracy, have been used in archery to analyze the stability of the archer’s hands [7]. Based on signal features collected from 3D accelerometers, activities such as football, Nordic walking, and cycling were recognized [8]. Inertial sensors were attached to the upper arm to prevent shoulder injuries in baseball and volleyball [9]. The use of sensor technology and advanced signal processing techniques can improve player performance, help prevent injuries and save training time and costs.

Our motivation is to develop an augmented biofeedback system to accelerate motor learning and skill levels in precision shooting training. Our idea is to use pistol-mounted kinematic sensors [10] to measure hand movement, which is the most important human factor influencing the accuracy of precision shooting. Specifically, there are two main types of factors that influence the accuracy of precision shooting [11], (1) physical factors that can be controlled: including pistol parameters, precision shooting distance, weather, etc., and (2) human factors: including pistol grip, hand movement, aiming and trigger pull. Based on the measured hand movement signals and knowledge of the most common errors in precision shooting, we create a machine learning model to predict shooting accuracy and precision parameters [12]. Based on the proposed model, we have developed an augmented biofeedback system that provides concurrent and terminal feedback to users.

There has been some work related to the use of kinematic sensors in precision shooting. The authors of [13] developed a motion analysis system based on the technology of inertial sensors to estimate posture or micro-movements such as posture sway during precision shooting. A target system to support the shooter during aiming was presented in [14]. The relation between postural balance, rifle stability and precision shooting performance was discussed in [15]. None of the above mentioned papers consider the factors that influence the shooting results in precision shooting, or gives concurrent and terminal feedback within the same system. To the best of our knowledge, there have been few studies on the prediction of precision shooting results using sensor data and machine learning techniques. Although some work has been found in [16,17] on the use of machine learning algorithms to predict shooting results, these use only static data, such as the basic information of the athletes (gender, age, status and precision shooting mode), and not the motion data collected by the sensors. Consequently, they cannot implement an augmented real-time biofeedback system that provides concurrent feedback to users.

In this paper, we investigate the use of kinematic sensors to accelerate motor learning in precision shooting training. We have developed a shot accuracy prediction model of precision shooting based on the Random Forest (RF) [18] classification algorithm. Its hyperparameters are optimized by a Bayesian hyperparameter optimization method [19]. This model can be used in an augmented real-time biofeedback system that provides concurrent feedback to the user. Shooting precision can only be calculated after the shooting session, defined as a group of *N* shots, has been completed. Therefore, a shot precision model cannot be implemented in scenarios with concurrent feedback. In this article we focus primarily on the shot accuracy prediction model.

As the most important scientific contribution, this paper proposes a new applicable methodology for accuracy prediction in pistol shooting. This serves as a basis for augmented feedback and leads to improvements in shooting skill acquisition and performance. The proposed methodology is based on: (1) an intelligent processing scheme for shooting sport data, including data acquisition, data pre-processing, outlier removal and feature selection, that provides an understanding of how to better process and analyze shooting sport data collected by sensors. (2) A shot accuracy prediction model for precision shooting based on a RF classification algorithm that achieves a higher prediction accuracy than other existing reference models; the proposed model can be considered as a promising candidate solution for shooting accuracy prediction by using kinematic sensors, (3) some reliable results that clearly show that hand movement is negatively correlated with shooting accuracy, and (4) a designed augmented biofeedback system based on machine learning techniques that meets the demand for accuracy prediction in precision shooting training scenarios and the system that can be used in concurrent and terminal feedback scenarios.

Concurrent feedback can bring benefits to users. For example, if the system predicts a poor shot result (based on measured signals, i.e., hand movement), it warns the user, who is advised to suspend the current shot. Such an action reduces the number of poorly executed shots, resulting in faster motor learning and fewer rounds used during training, saving money and time. The terminal feedback can provide information about other errors, such as triggering errors (after each shot) or aiming errors (after each series of shots). Both biofeedback modes have the potential to improve precision shooting performance.

The paper is further structured as follows. Section 2 introduces the methodology, including the distributed architecture of the biofeedback system, the measurement of precise shooting performance, data acquisition and preprocessing, classification, Bayesian hyperparameter optimization, and biofeedback application. Section 3 presents experiments for performance evaluation and comparison. Finally, we draw conclusions in Section 4.

## 2. Methodology

### 2.1. Biofeedback System

Biofeedback is a method of body control that uses a variety of sensors to measure physiological and physical functions, parameters and activities of people that they are usually unaware of or cannot perceive with their senses [20]. A biofeedback system typically includes a sensor device to collect the sensor signals and data, a processing device to process the collected signals and data, wireless or wired communication channels to transmit the sensor signals, and a feedback device that provides information to the person attempting to respond to the received feedback information [21,22].

**Figure 1:**
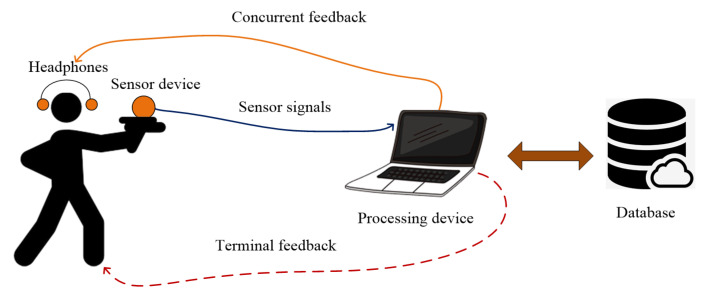
The distributed architecture of the biofeedback system. The acquired sensor signals are processed by the processing device. The user can receive concurrent feedback on unwanted or excessive hand movements (solid arrows) or terminal feedback on the shooting results (dashed arrow). All signals and processed data are stored in the database.

The distributed architecture of the augmented biofeedback system is shown in Figure 1. The system is designed to operate in two different modes: concurrent feedback and terminal feedback. In the concurrent feedback scenario, represented by solid arrows, the user receives audio feedback when an unwanted or excessive hand movement is detected. The user can decide to suspend the current shot and try again later. In the terminal feedback scenario represented by dotted arrows, the user receives feedback on performance. Such terminal feedback can contain information about many useful shooting parameters, such as triggering error, aiming error, shooting statistics and others.

### 2.2. Measurement of Precise Shooting Performance

**Figure 2:**
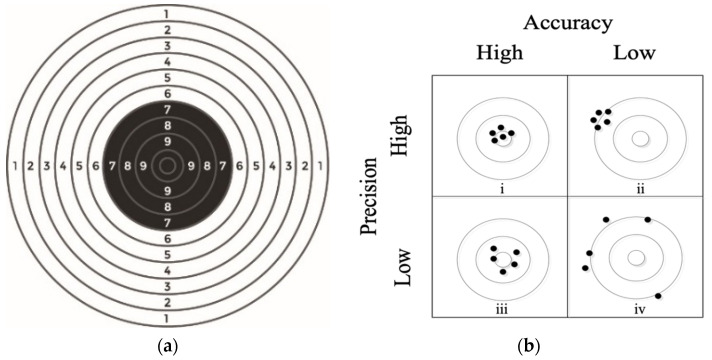
(**a**) Standardized pistol target; (**b**) the distribution of accuracy and precision: (i) high accuracy and high precision, (ii) low accuracy but high precision, (iii) low precision but high accuracy, (iv) low accuracy and low precision.

In precise shooting practice, shots are fired at the standardized pistol target shown in Figure 2a. The outer nine rings are 50 mm wide and the inner two rings are 25 mm wide. The results are scored according to the distance from the center from 0 (miss) to 11 (center). We define the shooting session as a group of *N* fired shots. Usually a shooting session consists of *N* = 5 shots. There are two basic measures for evaluating shooting performance: accuracy and precision, graphically shown in Figure 2b.

Accuracy refers to how well the shot impact point is centered on the standardized pistol target; the closer to the center of the target, the better the accuracy. The measures of shooting performance are the accuracy, i.e., the extent to which the center of the group of shots is close to the center of the target, and the precision, i.e., the tightness or the size of the group [12]. Precision describes the spread of individual shots around the center of the shot group; it should be noted that in this paper we define a group of 5 shots as one shooting session. An excellent shooter can achieve a high shooting result for all 5 shots if the shots are placed at a small distance from the center of the target. As already mentioned in the Introduction section, only shooting accuracy is discussed in this paper.

**Figure 3:**
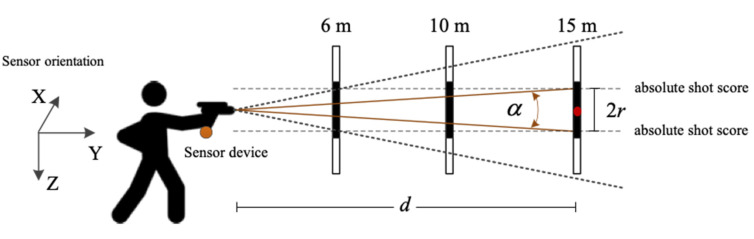
Precision shooting from three different distances including 6 m, 10 m and 15 m. Our sensor device mounted onto the bottom of the pistol grip can monitor the movement of the pistol in three dimensions.

The shot score, read from the target, is not a suitable measure of the shooting performance when we compare the results at different target distances [23]. The three most commonly used distances in shooting practice are 6 m, 10 m and 15 m, as shown in Figure 3. Obviously, it is much easier to achieve a good result at shorter distances than at longer distances. A relatively good shot at 6 m would be a poor shot at 10 m and a miss at 15 m, see the thick dotted lines in Figure 3.

In order to fairly evaluate the precision shooting performance from different shooting distances, we use the shot result angle as a measure instead of the absolute shot result in numerical values from 0 to 11, which are read from the target. The shot result angle is defined by Equation (1), where *r* is the distance between the point of shot impact and the center of the target and *d* is the shooting distance.

(1) $$\alpha =2\cdot \arctan\left(\frac{r}{d}\right)$$

In our research we define only two types of shot results: the good and the bad. The test subjects shoot from three different distances: 6 m, 10 m and 15 m. We define a good shot based on the angle α. The threshold value is defined according to the shot result of 8 at a shooting distance of 15 m, which gives $\alpha =0.5^{{}^{\circ}}$. For example, for the same shot angle α, the shot score can be classified as good (8) at 6 m, medium (4) at 10 m and bad (out of target) at 15 m shooting distance; see the dotted lines in Figure 3.

### 2.3. Data Acquisition and Preprocessing

**Figure 4:**
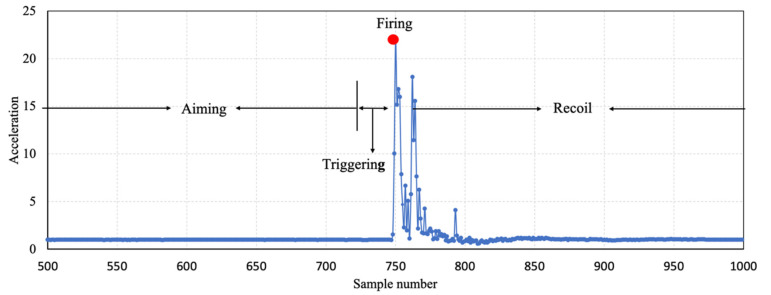
The absolute value of the 3D acceleration signal of a fired shot divided into four phases: aiming, triggering, firing and recoil.

Our sensor device mounted onto the bottom of the pistol grip includes a 3-axis accelerometer and a 3-axis gyroscope. The sampling frequency *f_s_* of our sensor device is 250 Hz and the number of recorded samples for each shot is 1000; 750 samples (3 s) are recorded before the firing and 250 samples (1 s) are recorded after the firing. Figure 4 shows the absolute value of the acceleration signal of a fired shot. For a better readability of the graph, only the signal between sample numbers 500 and 1000 is plotted. As shown in Figure 4, there is an evident change in acceleration at sample number 750, which represents the firing moment of the pistol. Each shot is divided into four phases: the aiming, triggering, firing and recoil phases. It was proved in [10] that the hand movement before the firing phase is strongly negatively correlated with the accuracy of the precision shooting.

**Figure 5:**
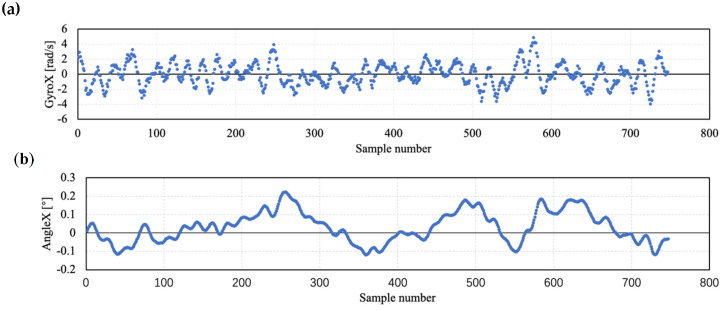
Measured and calculated sensor signals. (**a**) The measured angular velocity signal around X-axis, (**b**) the calculated angle signal around X-axis.

We can acquire the angular velocity signals ω[n] from the 3-axis gyroscope of the sensor device. The angular velocity signal around the X-axis is shown in Figure 5a. Since we use the shot angle result as a measure of performance, we use ω[n] to calculate the angle signal β[n] by the expression Equation (2), where $T_{s}$ is the sampling time. The angle signal β[n] is shown in Figure 5b, which is calculated from the angular velocity signal ω[n] from Figure 5a.

(2) $$\beta \left[n\right]=\sum_{i=0}^{i=n}\omega \left[i\right]T_{s}$$

In total, we have six measured signals: angular velocity (Gx,Gy,Gz) acquired by a 3-axis gyroscope, acceleration (Ax, Ay, Az) acquired by an accelerometer. The angle signals β (Ax, Ay, Az) are derived from angular velocity signals (Equation (2)). For each shot we also calculate an angle variation value (peak-to peak) in triggering phase, just before firing; as discussed in detail in Section 2.4.

### 2.4. Classification

In this paper, we perform a binary classification by defining only two types of shot results: the good and the bad, according to the shot angle threshold of 0.5° as defined by Equation (1). If the shot angle exceeds the threshold, the shot is classified as bad, otherwise it is classified as good. The movement of the pistol is acquired through the attached sensor device including a 3-axis accelerometer and a 3-axis gyroscope. The measure for pistol movement is the standard deviation of the measured and derived sensor signals; for example, the standard deviation of the rotational speed around the X-axis. We use the Pearson correlation coefficient method [24] and the importance score of each feature obtained by the random forest (RF) model to obtain the optimized feature set to train a model for model training.

**Figure 6:**
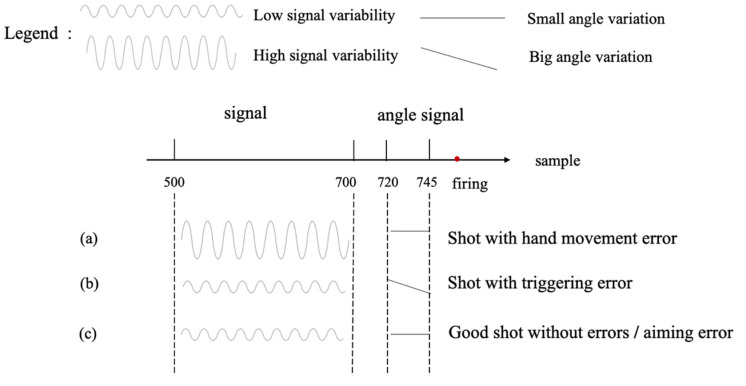
Illustration of the three most common errors in precision shooting: (**a**) hand movement error; (**b**) triggering error; (**c**) aiming error or a good shot.

To obtain the data of the training model, we have to predefine the conditions and measures for data cleaning and the removal of outliers. There are three basic types of errors in precision shooting that can influence the shot results: hand movement errors, aiming errors and triggering errors [11]. The hand movement error occurs when the pistol movement exceeds a predefined threshold, which is caused by the shooter’s conscious or unconscious body/arm swaying. We can detect this error by calculating the variability of the sensor signals, as shown in Figure 6a. We measure the variability of the signal values between sample numbers 500 and 700 to give the users enough time to react to possible feedback on the motion error. It usually takes about 180 ms for people to take action when they receive the feedback [25]. The triggering error occurs when the trigger is pulled incorrectly, resulting in an unwanted pistol movement just before the shot is fired. We can monitor this error by analyzing the change in the pistol angle in the last 100 ms before firing, in Figure 6b this happens at angle signals with sample numbers between 720 and 745. The aiming error occurs due to an incorrect aiming technique. It cannot be detected by analyzing sensor signals, but by statistically analyzing a number of shots from the same user after the shooting session. The aiming error scenario is shown in Figure 6c, which shows that the aiming error cannot be distinguished from the signal of a good shot.

Significantly, the hand movement before the firing phase is strongly negatively correlated with the accuracy of the precision shot, as demonstrated in [10]. However, there is one exception—where the shot result is good even if the hands move too much—we called this lucky shots.

**Figure 7:**
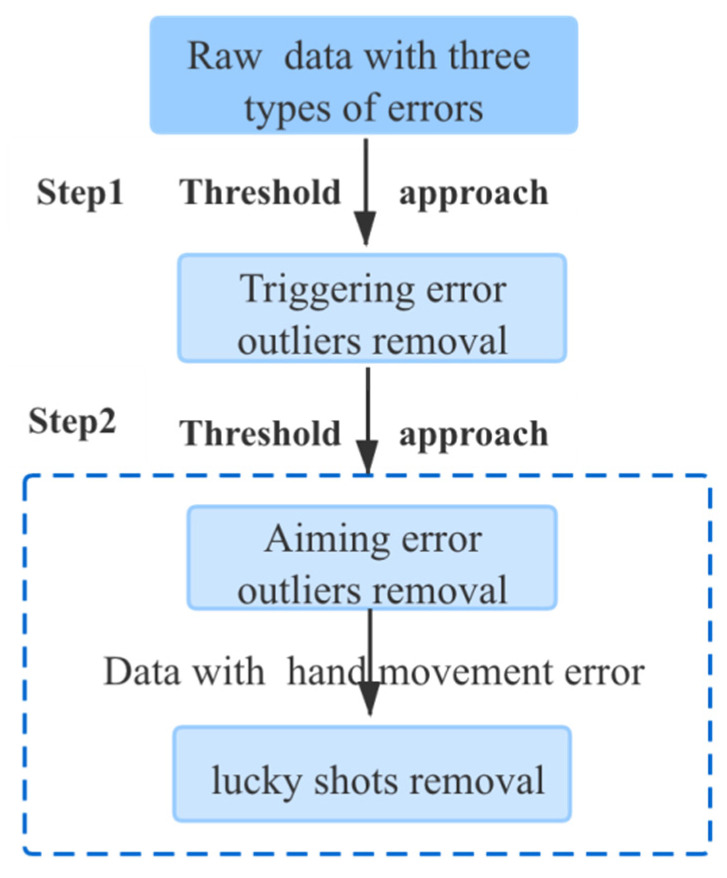
Outlier removal processing.

Our goal is to establish a model for hand movement error based on machine learning using the sensor signals and data. To achieve this, it is necessary to eliminate the interference of the other two errors—triggering error and aiming error as well as lucky shots. Any data that interferes with the model training for a just reason are defined as outliers. The outlier elimination flowchart is shown in Figure 7.

First, we have to identify and eliminate the triggering error outliers. The triggering error typically occurs within the last 100 ms (25 samples) before the firing phase, so we analyze all signals between sample numbers 720 and 745. The guard interval of the trigger phase is 5 samples (20 ms). In most cases the triggering error causes a vertical and/or lateral movement of the gun. The expression for the detection of a triggering error is defined by Equation (3), where *R* is the difference between the maximum and minimum value of β[n] where the value of *n* is a sample number between 720 and 745. The shot score is influenced by R; if R is greater than the predefined score shot angle $\alpha =0.5^{{}^{\circ}}$, a triggering error is very likely. For this reason, we define the threshold value for the triggering error angle at $R>0.5^{{}^{\circ}}$.

(3) R=Max(β[n])−Min(β[n]),720≤n≤745

**Figure 8:**
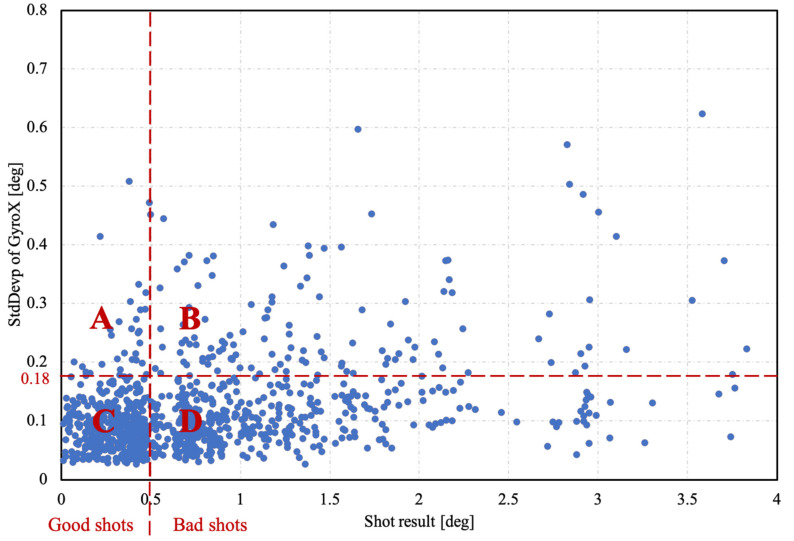
Detection of outliers denoted as lucky shots and the shots with aiming error. Area A is the area of lucky shots, the points in Area B and C represent the hand movement detection regions, and Area D most probably is the area denoting points of aiming error.

After we have eliminated the triggering error outliers, we detect and remove some other known outliers. The most important datum collected by the sensors is the angular velocity (ω[n]) around the X-axis, since the movement of the pistol most is often in a vertical and lateral direction. Therefore, we focus primarily on the gyroscope signals around the X-axis. In Figure 8, the standard deviation of the pistol angle around the X-axis, calculated from the β[n] signal, is plotted against the shot result expressed by its angle. The threshold value for the shot result is 0.5°, which was defined to distinguish between good and bad shots.

As can be seen in Figure 8, there is another threshold value, which is defined at 0.18° of the standard deviation of the pistol angle around the X-axis. In [10], the authors have shown that hand movement and shot score are negatively correlated; higher values of hand movement lead to a lower shot score and consequently to a higher shot angle. The current study has established that more experienced shooters have smaller shot angles, as shown in Section 3. Based on the above, it is unlikely that a high hand movement will give a good shot score, but in some cases, it happens—we call such shots lucky shots. Based on our experience with shooting tests, we set the estimated probability of lucky shots at 5%. Under this assumption, we calculate the threshold value of 0.18°, which meets our criterion. In order to validate our assumption, our system should be supplemented by an optical measuring system, which was not within the scope of our study.

Both thresholds divide the graph into four areas. The shots in area A are lucky shots. Area B and area C correspond to the conclusions in [10], where the precision shooting result is inversely proportional to the standard deviation of the hand accelerations and rotations. Area D most likely contains the shots with the aiming error, because there must be no triggering errors and the hand movement is also small, but the shot result is still bad.

Using the above method, we can remove shots with aiming errors, triggering errors and lucky shots. After this procedure we can successfully establish the accuracy prediction model of precision shooting based on the random forest.

The random forest (RF) is an ensemble tree-based algorithm, which is specified as follows:

(a) Utilize bagging to randomly generate *k* diversified subsets ($D_{1,}D_{2},D_{3},\ldots ,D_{k}$) of the entire training set D.
(b) For each subset $D_{i}$, grow an unpruned classification tree $T_{i}$. During the splitting of each node, rather than choosing the best split among all predictors M, randomly selects $m_{try}$ ($m_{try}\ll M$) of these predictors M, and then choose the best split among those variables.
(c) Predict new data by aggregating the predictions of the k trees $T_{k}$ following the majority decision rule.

### 2.5. Bayesian Hyper-Parameter Optimization

The classification algorithms usually have several hyperparameters that strongly influence the accuracy of the model. The most common methods for selecting hyperparameters are mainly Grid Search [26], Random Search [27] and Bayesian Optimization. Among them, Grid Search, also known as brute force search method, is currently the most common one. It runs through the hyperparameter combination in a loop, searching for all values in the range of candidate parameters and uses the most powerful parameters as optimal parameters of the model. Random Search does not loop through all candidate parameter values, but randomly selects a fixed number of parameters from the given parameter distribution. The disadvantage of these two methods is that each new estimate of the optimal parameter is independent of the previous training. If the amount of data is large, many parameters must be tuned, which requires excessive computational resources.

**Figure 9:**
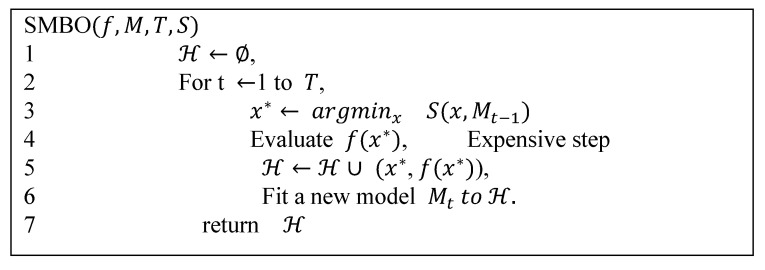
The pseudo-code of sequential model-based optimization, where f is the objective function, M is a cheaper-to-evaluate surrogate model, S is an auxiliary criterion function.

Bayesian optimization [19], which achieves state-of-the-art results for a few global optimization problems, is an effective solution for hyperparameter tuning. The application scenario of the Bayesian optimization method is that only the input and output values are known while the function equation, function structure and the mathematical properties are unknown. Known points are used to learn the form of the objective function. The specific method consists of increasing the sample points and updating the posterior probability distribution of the objective function until the posterior distribution is basically consistent with the real distribution. Compared to grid and random search, it is better to use the last parameter information to adjust the current parameters. In this paper, we use a sequential model-based optimization (SMBO) algorithm [28], which is an advanced Bayesian optimization scheme for tuning the hyperparameters. SMBO usually builds a model to select the optimal hyperparameters in a defined search space, and is particularly common when evaluating the cost function is expensive. The SMBO algorithm is shown in Figure 9.

### 2.6. Biofeedback Application

The methods described can be used in a biofeedback application as follows:

(a) The accuracy prediction model, gives the user concurrent feedback on hand movement error when working in a real-time scenario; if the result of the prediction is a bad shot, the application advises the user to suspend the shot, calm down and try again later;
(b) After each shot, the application checks for possible triggering errors in post-processing mode and gives the terminal feedback to the user;
(c) At the end of the shooting session, the application calculates the statistical values of precision and accuracy and provides terminal feedback about possible aiming errors.

## 3. Experiments and Results

### 3.1. Experimental Setup

The collection of the shooting results was carried out with the help of 61 subjects, of different ages, genders and shooting skill level. The total sample of respondents included beginners, intermediate, experienced, professional and sport shooters. The majority of shooters were men (43), while 18 shooters were women. Shootings were carried out in five measurement sessions on different dates. Descriptive statistics, including mean value, standard deviation, coefficient of variance (CV), maximum and minimum shot angle, are presented for each session and skill level group in Table 1 and Table 2, respectively.

**Figure 10:**
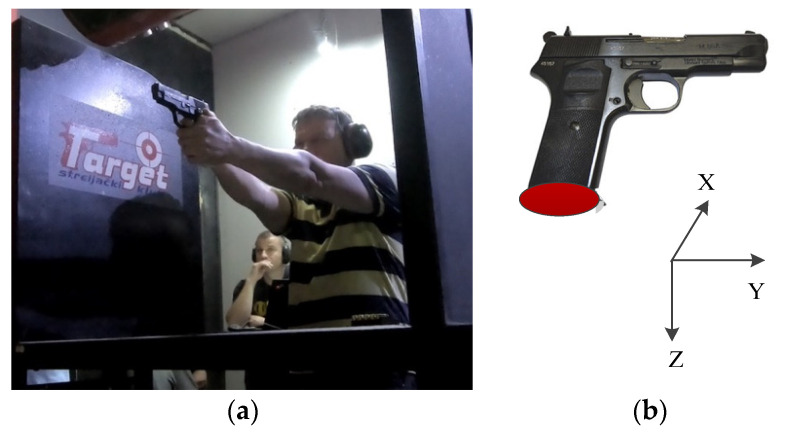
Precise shooting measurements: (**a**) in our precision shooting experiment, trainees need to use both hands to support the pistol model; (**b**) the orientation of the sensor coordinate system.

The shooters used both hands to support the CZ99 pistol model, as shown in Figure 10a. The placement of the sensor device and the orientation of the sensor coordinate system is shown in Figure 10b. The shooters conducted 193 shooting sessions, with each session comprising 5 shots, so that a total of 965 shots were fired. Shots were fired from distances of 6 m, 10 m and 15 m. The shot result angle was used as a performance measure at all distances. The shot results are divided into two groups, the good and the bad, according to the shot angle threshold of 0.5°. If the shot angle exceeds the threshold value, the shot result is bad, otherwise it is good. We use 1 and 0 to mark the good and bad results.

The initial feature set includes the standard deviation of the angular velocity signal (StdGx, StdGy, and StdGz), the standard deviation of the angle signal β (StdAx, StdAy, StdAz), the standard deviation of the acceleration signal (StdAccx, StdAccy, StdAccz), and the angle range (Rx, Ry, and Rz) defined as the difference between the maximum and minimum of β from the sample before 700 for three dimensions. Combining these 12 features with a shot result finally yields a data set with a matrix size of 965 × 13.

After we have eliminated the outliers, we randomly shuffle the remaining data set (720 × 13) and randomly split it into a training set and a test set with a ratio of 8:2. The training set is used to build the RF-based accuracy prediction model of precision shooting, whose parameters are selected by 10-fold cross-validation and Bayesian hyperparameter optimization. The test set is also used for evaluation. The classification algorithms used in this paper are trained with the Scikit learning package of the Python software. Bayesian hyperparameter optimization is performed using the hyperopt [29] package for Python.

### 3.2. Feature Selection

In order to obtain the key features related to the performance of precision shooting, we carry out a feature selection. First, we calculate the Pearson correlation coefficient [24] for each feature and the corresponding shot result. Table 3 shows that the results of all features are negative, indicating that the greater the values of all features, the worse the shot result. It also proves that the hand movement before the firing phase is strongly related to the accuracy of the precision shooting [10].

**Figure 11:**
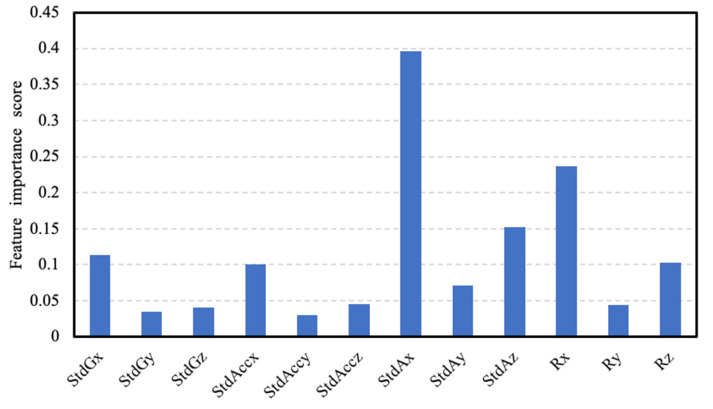
The feature importance scores.

Next, we use the initial feature set to build a model based on RF. The scores of the feature importance are shown in Figure 11. The variability of the β[n] of the X-axis achieves the highest score, which proves that the angle feature is strongly related to the shot result and the movement of pistol often appears in the upward direction (X-axis), as mentioned in Section 2. The reason why the variability of β[n] can achieve a higher score than the angle range is that it can better reflect the hand movement of changes before firing.

Finally, we use the Embedded method which is performed by Select From Model function [30] in the feature selection module of scikit-learn. The selected estimator is RF and the threshold is set to 0.1, so the remaining features set are StdGx, StdAccx, StdAx, StdAz, Rx and Rz.

### 3.3. Algorithm Testing and Selection

**Figure 12:**
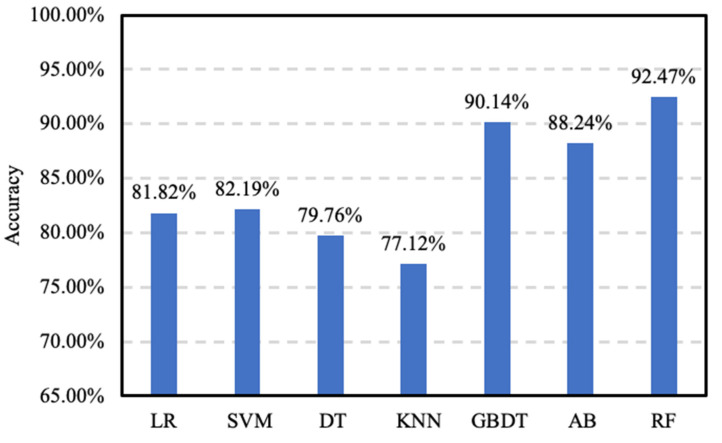
The accuracy of different classification algorithms on the training set.

To evaluate the performance of the proposed accuracy prediction model optimized with Bayesian hyperparameter optimization, it is compared with a number of baseline models, including logistic regression (LR) [31], support vector machine (SVM) [32], decision tree (DT) [33], k-nearest neighbors (KNN) [34], random forest (RF) [18], Adaboost (AB) [35] and gradient boosting decision tree (GBDT) [36]. Because of an insufficient amount of data, deep learning methods cannot be used. After the 10-fold cross-validation and Bayesian hyperparameter optimization on the training set, we can determine the optimal parameters for the models. The results of the 10-fold cross-validation of the models with the optimal parameters are shown in Figure 12.

**Figure 13:**
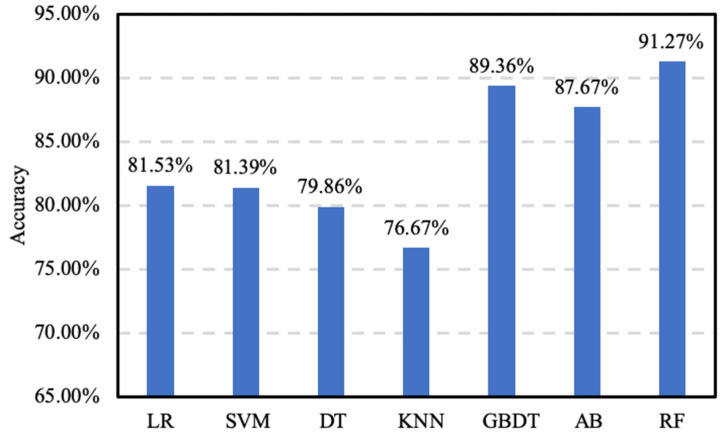
The accuracy of different classification algorithms on the test set.

Next, we evaluate the performance of the models with the optimal parameters on the test set. The results are shown in Figure 13, the RF-based accuracy prediction model achieves an accuracy of 91.27%, which outperforms all baseline models. GBDT is the best baseline model with the second highest accuracy of 89.36% and AB is close to GBDT. The result shows that ensemble models including RF, AB and GBDT perform better than the individual models including LR, SVM, DT, and KNN. The latter are simple algorithms that are widely used in machine learning, but suffer performance losses due to the overfitting problem. GBDT is easier to learn than RF because GBDT learns the residuals from the previous trees, and RF follows the majority decision rule. Many results are combined to give good generalization and higher accuracy.

In addition to accuracy, a number of other criteria for classification performance such as precision, recall and $F_{1}$ score are frequently used. We use these criteria to further evaluate the performance of each classification method, the results of which are presented in Table 4. The $F_{1}$ score is the harmonic mean of precision and recall. The higher the $F_{1}$ score, the more robust the classifier.

**Figure 14:**
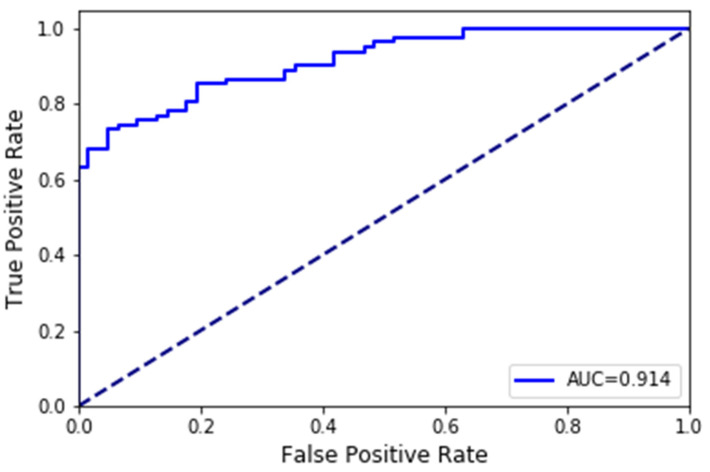
The ROC of RF.

The ROC (Receiver Operating Characteristics) curve of the proposed RF model is shown in Figure 14, which is one of the important evaluation metrics for testing the performance of the classification model. AUC (Area Under the Curve) represents the separability of the measure. The higher the AUC, the better the performance of this classifier. An excellent model usually has an AUC that is close to 1, which means it has a good measure of separability. On the contrary, a poor model has an AUC that is close to 0, which means that it has the worst performance. As shown in Figure 14, the AUC of the proposed RF model is 0.914, which proves that it is effective.

## 4. Conclusions

This paper proposes an accuracy prediction model of precision shooting based on the RF algorithm. Experimental results prove that the proposed model with RF achieves higher prediction accuracy than the other existing reference models. The results also prove that the hand movement is negatively correlated with the accuracy of precision shooting and that the angle feature is more relevant to the shot result than the angular velocity feature.

Based on this model, a biofeedback application is designed that operates in concurrent feedback mode and terminal feedback mode. If such a system detects excessive pistol movement and the proposed model provides a prediction that the result will be poor, the system gives the user a concurrent feedback and suggests that the user suspend the current shot and try again later. Such training can lead to a reduced number of poorly executed shots and consequently saving rounds, money and time. In addition, the system can provide terminal feedback after each shot, informing users and trainers of their errors and performance.

There is still a lot of work to be done in the future. The first step is to collect more data, including many more subjects and shooting sessions from all distances (6 m, 10 m and 15 m). With enough data we should be able to achieve higher prediction accuracy, the model of shooting precision could be improved and deep learning methods could be applied. The next steps we are planning are to test a method for reliable threshold determination for more accurate outlier detection and to build a multi-classification accuracy prediction model of all eleven shot scores.

## Figures and Tables

**Table 1:** The statistical parameters of shot angle of five measurement sessions (deg).

Measurement Session	No. of Shots	Mean	StDev	CV	Max	Min
1	200	1.39	1.38	1.00	7.16	0.02
2	170	1.23	1.15	0.94	7.39	0.02
3	180	1.22	1.37	1.13	7.40	0.04
4	210	1.48	1.48	1.00	7.39	0.03
5	205	1.26	1.20	0.95	7.33	0.01

**Table 2:** The statistical parameters of shot angle of five shooting skill level groups (deg).

Skill Level	Mean	StDev	CV	Max	Min
Beginner	1.65	1.45	0.88	7.40	0.03
Intermediate	1.01	0.73	0.72	4.19	0.02
Experienced	0.98	0.64	0.65	3.15	0.10
Professional	0.60	0.49	0.82	4.42	0.01
Sportist	0.21	0.11	0.53	0.38	0.01

**Table 3:** Pearson correlation between features and the shot result.

	**StdGx**	**StdGy**	**StdGz**	**StdAx**	**StdAy**	**StdAz**
Result	−0.46	−0.41	−0.40	−0.54	−0.54	−0.30
	**StdAccx**	**StdAccy**	**StdAccz**	**Rx**	**Ry**	**Rz**
Result	−0.52	−0.30	−0.05	−0.51	−0.31	−0.04

**Table 4:** The results of three criteria of different classifiers.

	LR	SVM	DT	KNN	GBDT	AB	RF
Recall	0.90	0.97	0.83	0.86	0.88	0.87	0.90
Precision	0.82	0.77	0.83	0.79	0.93	0.89	0.96
$F_{1}$ score	0.86	0.86	0.83	0.82	0.90	0.88	0.93

## References

[1] Vanrell S.R., Milone D.H., Rufiner H.L. (2017). Assessment of homomorphic analysis for human activity recognition from acceleration signals. IEEE J. Biomed. Health.

[2] Duncan M.J., Roscoe C.M., Faghy M., Tallis J., Eyre E.L. (2019). Estimating physical activity in children aged 8-11 years using accelerometry: Contributions from fundamental movement skills and different accelerometer placements. Front. Physiol..

[3] Jiao L., Bie R., Wu H., Wei Y., Kos A., Umek A. (2018). Golf Swing Data Classification with Deep Convolutional Neural Network. IPSI BGD Trans. Internet Res..

[4] Kidman E.M., D’Souza M.J.A., Singh S.P.N. A wearable device with inertial motion tracking and vibro-tactile feedback for aesthetic sport athletes Diving Coach Monitor. Proceedings of the 2016 10th International Conference on Signal Processing and Communication Systems (ICSPCS).

[5] Wang J., Chen Y., Hao S., Peng X., Hu L. (2019). Deep learning for sensor-based activity recognition: A survey. Pattern Recognit. Lett..

[6] Aroganam G., Manivannan N., Harrison D. (2019). Review on wearable technology sensors used in consumer sport applications. Sensors.

[7] Loke Y.L., Gopalai A.A., Khoo B.H., Senanayake S.M.N.A. Smart system for archery using ultrasound sensors. Proceedings of the 2009 IEEE/ASME International Conference on Advanced Intelligent Mechatronics.

[8] Ermes M., Pärkkä J., Mäntyjärvi J., Korhonen I. (2008). Detection of daily activities and sports with wearable sensors in controlled and uncontrolled conditions. IEEE Trans. Inf. Technol. Biomed..

[9] Rawashdeh S., Rafeldt D., Uhl T. (2016). Wearable IMU for shoulder injury prevention in overhead sports. Sensors.

[10] Kos A., Umek A., Marković S., Opsaj M. (2019). Sensor System for Precision Precision shooting Evaluation and Real-time Biofeedback. Procedia Comput. Sci..

[11] Yang C.C., Hsu Y.L. (2010). A review of accelerometry-based wearable motion detectors for physical activity monitoring. Sensors.

[12] Johnson R.F. (2001). Statistical Measures of Marksmanship.

[13] Dinu D., Fayolas M., Jacquet M., Leguy E., Slavinski J., Houel N. (2016). Accuracy of postural human-motion tracking using miniature inertial sensors. Procedia Eng..

[14] Osborn J. (2004). Method and apparatus to provide precision aiming assistance to a shooter. U.S. Patent.

[15] Sattlecker G., Buchecker M., Müller E., Lindinger S.J. (2014). Postural balance and rifle stability during standing precision shooting on an indoor gun range without physical stress in different groups of biathletes. Int. J. Sports Sci. Coaching.

[16] Deng S., Liu D.M., Hsieh S.L. (2011). Applying machine learning methods to the precision shooting accuracy prediction: A case study of T-75 pistol precision shooting. Inf. Technol. J..

[17] Maier T., Meister D., Trösch S., Wehrlin J.P. (2018). Predicting biathlon precision shooting performance using machine learning. J. Sports Sci..

[18] Elola A., Aramendi E., Irusta U., Del J., Alonso E., Daya M. (2019). ECG-based pulse detection during cardiac arrest using random forest classifier. Med. Biol. Eng. Comput..

[19] Xia Y., Liu C., Li Y., Liu N. (2017). A boosted decision tree approach using Bayesian hyper-parameter optimization for credit scoring. Expert Syst. Appl..

[20] Kos A., Anton U. (2018). Biomechanical Biofeedback Systems and Applications.

[21] Matevž H., Anton U., Anton K. (2020). Survey of recent development in real-time biofeedback systems in sport. Serbian J. Sports Sci..

[22] Zhang Y., Umek A., Obinikpo A.A., KOS A. A Time-Dependent Multi-Class SVM Algorithm for Crowdsourced Mobility Prediction. http://ipsitransactions.org/journals/papers/tir/2018jan/p7.pdf.

[23] Dopsaj M., Markovic S., Umek A., Prebeg G., Kos A. (2019). Mathematical model of short distance pistol shooting performance in experienced shooters of both gender. NBP Nauka Bezbednost Policija.

[24] Lawrence I., Lin K. (1989). A concordance correlation coefficient to evaluate reproducibility. Biometrics.

[25] Kosinski R.J. (2008). A literature review on reaction time. Clemson Univ..

[26] Probst P., Wright M.N., Boulesteix A.L. (2019). Hyperparameters and tuning strategies for random forest. Wiley Interdiscip. Rev..

[27] Bergstra J.S., Bardenet R., Bengio Y., Kégl B. Algorithms for hyper-parameter optimization. Proceedings of the Advances in Neural Information Processing Systems.

[28] Bardenet R., Brendel M., Kégl B., Sebag M. Collaborative hyperparameter tuning. Proceedings of the International conference on machine learning.

[29] Bergstra J., Komer B., Eliasmith C., Yamins D., Cox D.D. (2015). Hyperopt: A python library for model selection and hyperparameter optimization. Comput. Sci. Discov..

[30] Pedregosa F., Varoquaux G., Gramfort A., Michel V., Thirion B., Grisel O., Blondel M., Prettenhofer P., Weiss R., Dubourg V. (2011). Scikit-learn: Machine Learning in Python. J. Mach. Learn. Res..

[31] Hosmer D.W., Lemeshow S., Sturdivant R.X. (2013). Applied Logistic Regression.

[32] Choubin B., Moradi E., Golshan M., Adamowski J., Sajedi-Hosseini F., Mosavi A. (2019). An ensemble prediction of flood susceptibility using multivariate discriminant analysis, classification and regression trees, and support vector machines. Sci. Total Environ..

[33] Safavian S.R., Landgrebe D. (1991). A survey of decision tree classifier methodology. IEEE Trans. Syst. Man Cybern..

[34] Cover T., Hart P. (1967). Nearest neighbor pattern classification. IEEE Trans. Inf. Theory.

[35] Schapire R.E. (2013). Explaining Ada Boost.

[36] Friedman J.H. (2001). Greedy function approximation: A gradient boosting machine. Ann. Stat..

